# Alcohol Use Disorder among Patients Suffered from Road Collisions in a Vietnamese Delta Province

**DOI:** 10.3390/ijerph16132423

**Published:** 2019-07-08

**Authors:** Hai Minh Vu, Tung Thanh Tran, Giang Thu Vu, Cuong Tat Nguyen, Chau Minh Nguyen, Linh Gia Vu, Tung Hoang Tran, Bach Xuan Tran, Carl A. Latkin, Cyrus S.H. Ho, Roger C.M. Ho

**Affiliations:** 1 Department of Trauma, Thai Binh University of Medicine and Pharmacy, Thai Binh 410000, Vietnam; 2 Center of Excellence in Evidence-based Medicine, Nguyen Tat Thanh University, Ho Chi Minh City 700000, Vietnam; 3 Institute for Global Health Innovations, Duy Tan University, Da Nang 550000, Vietnam; 4 Institute of Orthopaedic and Trauma Surgery, Vietnam—Germany Hospital, Hanoi 100000, Vietnam; 5 Institute for Preventive Medicine and Public Health, Hanoi Medical University, Hanoi 100000, Vietnam; 6 Bloomberg School of Public Health, Johns Hopkins University, Baltimore, MD 21205, USA; 7 Department of Psychological Medicine, National University Hospital, Singapore 119074, Singapore; 8 Center of Excellence in Behavioral Medicine, Nguyen Tat Thanh University, Ho Chi Minh City 700000, Vietnam; 9 Department of Psychological Medicine, Yong Loo Lin School of Medicine, National University of Singapore, Singapore 119228, Singapore; 10 Institute for Health Innovation and Technology (iHealthtech), National University of Singapore, Singapore 119077, Singapore

**Keywords:** traffic accidents, alcohol, drink-driving, Vietnam

## Abstract

Traffic collisions have continuously been ranked amongst the top causes of deaths in Vietnam. In particular, drinking has been recognized as a major factor amplifying the likelihood of traffic collisions in various settings. This study aims to examine the relationship between alcohol use and traffic collisions in the current context of Vietnam. A cross-sectional study was conducted on 413 traffic collisions patients in six health facilities in the Thai Binh Province to investigate the level of alcohol consumption and identify factors influencing alcohol use among these patients. The Alcohol Use Disorders Identification Test-Consumption (AUDIT-C) scale was used to determine the problematic drinking behavior of the participants. The percentage of patients having problematic drinking was more than 30%. Being male, having a high household income, and working as farmer/worker were risk factors for alcohol abuse. People causing accidents and patients with a traumatic brain injury had a higher likelihood of drinking alcohol before the accidents. This study highlights the necessity of more stringent laws on reducing drink-driving in Vietnam. In addition, more interventions, especially those utilizing mass media like educational campaign of good behavior on social networks, are necessary to reduce alcohol consumption in targeted populations in order to decrease the prevalence and burden of road injuries.

## 1. Introduction

Traffic collisions, a major public health problem, has been worsening in recent years. According to the World Health Organization (WHO), 20–50 million people suffer from road collisions each year [1]. Moreover, road traffic injuries (RTIs) have been recognized as the main cause of death globally [2], with the number of deaths due to RTIs increasing from 1.2 million in 2012 to 1.35 million in 2016 [3,4]. Most of the deaths due to RTIs have occurred in low and middle-income countries, especially in Africa and South East Asia [2,4]. The global status report on road safety 2018 of WHO indicated that the majority of traffic collisions deaths in South East Asia belonged to people using two and three-wheelers, which are common vehicles among South East Asian countries, including Vietnam [3,5].

Traffic collisions are continuously ranked amongst the top causes of death in Vietnam, a low-middle income country [6,7,8]. According to the General Statistics Office of Vietnam, in the first three months of 2019, there were a total of 4030 road traffic collisions which led to the death of 1905 people [7]. There were approximately 21 road crashes every day, resulting in 21 deaths, 13 people suffering from severe injuries, and 22 people having other injuries [7]. Additionally, motorcycles, the most popular means of transportation in Vietnam, have been demonstrated to be more dangerous than other types of vehicles [5]. Thus, more attention is required to reduce the number of road accidents in Vietnam.

As a cultural driver of risk behavior, drinking has been recognized as a major cause of road collisions in various settings [4,9]. Driving after consuming alcohol significantly increases the probability of road crashes in all ages [4,9]. A study revealed that patients in road collisions related to alcohol were more likely to suffer severe injury [10]. Drinking alcohol before driving was also found to have a great contribution to the number of road traffic deaths [11]. It is estimated that about 35% of RTI deaths are related to alcohol drinking globally [4]. People who consumed alcohol before driving are more likely to drive at a higher speed and have a slower reaction time [12], both of which result in serious RTIs and fatalities—not only to drivers but also to passengers and pedestrians. The relationship between alcohol consumption levels and different types of injuries has been well-documented in many studies [13,14,15]. However, alcohol consumption behavior is diverse in many countries or cultures [16]. Therefore, there is a need for investigating the relationship between alcohol consumption and different types of injuries in Vietnam, where the evidence of this topic is limited.

Though drink-driving laws have been applied in many countries in the world [4], those laws only have positive effects on reducing the use of alcohol before driving in high-income countries [6]. In Vietnam, the government promulgated drink-driving laws in 2008 [17]. It is compulsory that the blood alcohol concentration (BAC) level does not exceed 50 milligrams in 100 milliliters of blood or 0.25 milligrams in 1 L of breath [18]. People who do not obey the law may have to pay $86–774 depending on their BAC level [18]. However, most of road collisions have still been reportedly related to alcohol drink before driving [19]. Therefore, in order to heighten and enhance the effects of drink-driving laws, there is a need to investigate the factors associated with drink-drinking in Vietnam, where this problem still widely exists [6].

While the need for deeper evidence about alcohol consumption and traffic collisions is high in Vietnam, there is a lack of data in the literature about this topic. With the aim of addressing the issue, this study was conducted in six health facilities in the Thai Binh province, Vietnam, to investigate the level of alcohol consumption among traffic accident patients and its associated factors.

## 2. Materials and Methods

### 2.1. Study Setting

A cross-sectional study was conducted in 2018 at the Trauma—Orthopedic/Burn and Surgery department of the Thai Binh Hospital and five district-level hospitals in the Thai Binh province: Kien Xuong, Hung Ha, Dong Hung, Quynh Phu, and Thai Thuy. The convenience sampling method was used to recruit participants. The eligibility criteria for choosing participants were: (1) Being 18 years old or above; (2) suffering from trauma after a road crash; (3) currently receiving treatments at mentioned hospitals; and (4) being able to answer the interviewers (doctors and nurses at the departments). Interviewers were trained by experts to ensure the quality of data. Interviews took place in a private area to ensure confidentiality. Patients who met the eligibility criteria were invited to participate in the study. The interviewers clearly explained the aim of this study and ensured that patients’ privacy information would be encoded and only used for research purpose. After that, participants were requested to give the verbal informed consent. There was a total of 430 patients approached by interviewers, and 413 agreed to take part in the study.

### 2.2. Measurements and Instruments

We collected data via face-to-face interviews, which lasted 10–15 min, using a structured questionnaire. Interviewers were doctors or nurses from the above-mentioned hospitals. We collected data about social-demographic characteristics, types of injuries, their current treatments, and details about alcohol consumption.

#### 2.2.1. Social-Demographic

The social-demographic characteristics considered were age, gender, education, marital status, occupation, and living area. We also asked participants whether they were the person who caused the traffic collision (drivers), the person who got hit by other vehicles, the pedestrians or passengers (victim), or the person who was driving alone and crashed into some objects such as a tree, building, or pavement (self-accident).

#### 2.2.2. Injury Characteristics

We asked patients whether they suffered from wounds/damages after the collisions, such as a flesh wound, hand wound, traumatic brain injury, maxillofacial injury, spine injury, chest injury, fracture, or flesh injury.

#### 2.2.3. Alcohol Consumption

The level of alcohol consumption and problematic drinking were graded using the Alcohol Use Disorders Identification Test-Consumption (AUDIT-C). The AUDIT-C scale has been used in many studies in Vietnam [20,21]. The AUDIT-C scale is a brief measurement with 3 questions to screen problematic drinking of respondent: (1) How often do you have a drink containing alcohol? (2) How many standard drinks containing alcohol (30 mL/cup of wine or 1 can of beer) do you have on a typical day? (3) How often do you have six or more drinks (180 mL wine or 6 cans of beer) on one occasion? A total score of the AUDIT-C scale ranged from 0 to 12. The higher the AUDIT-C score was, the more likely the participants were to have problematic drinking. A hazardous drinking group was defined as the total score higher than 4 for males and higher than 3 for females. A binge drinking group was defined as drinking 6 or more drinks (cup or can) on one occasion [20,21]. Moreover, we also asked the patients about whether they consumed any drink containing alcohol within 12 h before the traffic collisions.

### 2.3. Statistical Analysis

Data were analyzed by STATA version 14.0 (Stata Corp. LP, College Station, United States of America). Frequency (%) was used to present categorical variables. Mean and Standard Deviation (SD) were used to interpret numerical variables. We utilized the chi-squared and Mann–Whitney tests to analyze the differences between the proportions of hazardous drinking and non-hazardous-drinking group; binge drinking and non-binge-drinking groups; and a group of patients who drank before the collisions and those did not. Since problematic drinking (binge and hazardous drinking) and consumed alcohol within 12 h before the traffic collisions characteristic are binary variables, we used multivariate logistic regression to identify the associated factors. In addition, to find the factors that had an association with an AUDIT-C score, we applied multivariate Tobit regression. We also applied a forward stepwise selection strategy to remove insignificant variables, and the threshold to select variables for reduced models was 0.2 of the log likelihood. A *p*-value < 0.05 was considered as statistical significant.

### 2.4. Ethics Approval

The study protocol was reviewed and granted ethics approval by the Institutional Review Board of the Thai Binh General Hospital.

## 3. Results

Among 413 patients taking part in the study, the mean age was 45.5 (SD = 17.0) years old, and 61.3% of them were female. Most of the respondents worked as a farmer or worker (55.0%), received secondary school education (35.1%), lived in the urban area (85.5%), had a spouse or partner (72.4%), and had an average household income per month of 8811.9 (SD = 5325.9) thousand VND. More than half of the respondents were victims of accidents (54.2%). (Table 1)

Table 2 shows that 56.4% of the patients never consumed any drink that contained alcohol. The proportion of people who only drank 1–2 cups each time, engaged in binge drinking, had hazardous alcohol according to the Audit scale and drank before the accidents were 70.7%, 36.6%, 30.5%, and 17.4%, respectively.

Table 3 shows the proportion of each injury type and the treatment among patients with different alcohol consumption characteristics. In terms of traumatic brain injuries and maxillofacial injuries, the proportions of patients who had binge drinking or consumed alcohol before the collisions were higher than those did not. In those who underwent osteosynthesis, the number of patients having problems with hazardous drinking was statistically significantly higher compared to other groups (*p*-value = 0.04). Meanwhile, a higher percentage of non-binge drinking patients was found in those who received the correction/bundle.

Table 4 indicates various factors associated with alcohol abuse among participants. Being male and having a higher household income were the risk factors for alcohol abuse. However, people who had a moderate household income were more likely to report a lower Audit score than those having the lowest household income. Students tended to have a lower Audit score (coefficient: −3.60; 95%CI: −6.17; −1.02) than freelancers. The risk of hazardous drinking in people who were living with their spouse or partner was about 2.61 times (OR: 2.61; 95%CI: 1.40; 4.89) higher than that of single people.

## 4. Discussion

This study investigated the prevalence and level of alcohol consumption among patients with RTIs using the AUDIT-C scale. The rate of binge drinking and hazardous drinking among participants was more than 30%. In addition, it was pointed out that socio–economic factors, such as gender, employment, and monthly household income, had an association with an AUDIT-C score, hazardous drinking, and binge drinking. Approximately one-fifth of patients were found to drink alcohol before their road crashes, while the multivariate logistic model revealed a relationship between traffic collisions and drinking alcohol within 12 h prior to the accidents. We also found that consuming alcohol within 12 h before an accident was positively associated with having a traumatic brain injury.

Among the 413 patients participating in this study, more than 30% were reported to be in binge drinking and hazardous drinking categories, and one fifth used alcohol before accidents. The prevalence of problematic drinking in our sample was higher than that of the general population in Vietnam [22]. These findings are consistent with other studies in various settings in RTI patients, which found the rate of alcohol consumption ranged from 24.4% to 30% [14,23,24]. The correlation between alcohol consumption and traffic collisions, which was found in this study, has also been reported in a number of studies [11,14,23]. In addition, traffic accidents related to alcohol consumption had a high prevalence of death [11,15,25]. A report by WHO indicated that 20% of deaths in traffic are related to alcohol consumption [26]. In order to ameliorate the current situation, this study also identified some factors associated with alcohol consumption in Vietnam. We found that socio–economic characteristics had an association with problematic drinking. Consistent with many studies in Vietnam, we found that females were less likely to use alcohol [27,28]. This may be explained by oriental culture, where drinking is usually considered as a means of communication among men [28]. The results also indicated that employment had an influence on alcohol consumption: Farmers and workers were more likely to have problematic drinking, as opposed to students. In addition, people with a higher income had a greater likelihood of engaging in binge drinking and hazardous drinking, a finding which is similar to a study in Vietnam by Kim et al. [29]. In Vietnam, there are a number of policies restricting alcohol consumption, such as the tax on alcohol productions and the national legal minimum age for alcohol customers [30], both of which cause difficulties for students purchasing alcohol products. In addition, patients with lower incomes may not be able to afford alcohol.

In terms of injury types, patients with hand wound were more likely to have lower alcohol consumption level. This might be explained by the fact that hand injuries reduce the ability to perform physical activities [31] and consuming alcohol impairs the progress of wound healing [32]. Therefore, the victims with this type of injury may avoid using alcohol and have a lower alcohol consumption level. Another noteworthy result is that respondents who consumed alcohol within 12 h before the crashes had a positive association with having traumatic brain injuries, which is important because brain injuries are the leading cause of disability. [33,34]. Since most of the Vietnamese population use motorcycles—which has been proven to be one of the major causes of traumatic brain injuries in traffic accidents [35]—as the principal means of transportation [5], the Vietnamese government promulgated a comprehensive motorcycle helmet policy in 2007 [36]. The policy has shown positive effects on reducing traumatic brain injuries by increasing the prevalence of using helmets [36]. However, people who used alcohol may lose the control of their actions [37], and, thus, exceeded the speed limit, lost driving control, and did not use protective gear such as helmets. Overall, these results provide more evidence to highlight the danger of traffic collisions related to alcohol, which is in alignment with previous studies [11,24,38]. Nevertheless, more studies are required to identify the precise associations between traffic collisions related to alcohol and other types of injuries in Vietnam.

Though Vietnam has promulgated laws to reduce alcohol-drinking, the results of this study reveal that drink-driving remains an urgent problem for the country, as the national alcohol consumption is increasing in recent years [28]. This requires more stringent laws from the government to reduce the prevalence and amount of drink-driving in Vietnam. Evidence has shown that lowering BAC-limits reduces RTIs and fatality rates [39]. In addition, tougher penalties such as increasing fines may help to reduce alcohol driving rates [40]. However, stringent laws are not the only solutions that can help to improve the drink-driving situation in Vietnam. With the proliferation of the internet and smart devices in Vietnam [41], smartphone and Internet-based interventions such as applications, which have been proven to be useful in reducing the alcohol consumption [42], can have a significant impact on improving the current situation. Interventions on social media, another method proven to effectively reduce drinking behavior [43,44], can help to reach a large amount of the population with diverse socio-economic characteristics. Moreover, using mass media as a tool to improve the knowledge of the negative effects of alcohol-driving may also be a feasible solution for the current context of Vietnam. Besides, increasing the tax and, consequently, the price of alcohol production could reduce the affordability of drinks that contain alcohol.

In this study, several limitations should be acknowledged. First, this study utilized a cross-sectional study design, which did not allow the team to determine the causal relationships between alcohol use and traffic collisions. Secondly, this study applied a convenient sampling method, resulting in the limited generalizability of the results. Therefore, this study was conducted in various settings. Thirdly, although the surveys were carried out by well-trained interviewers, recall bias may still have occurred due to the characteristics of self-report questionnaires. Fourth, the duration within which the use of alcohol products and traffic collisions reported in the questions was 12 h. This duration is higher than other studies [45,46]. However, we still found an association of consuming a drink containing alcohol within 12 h and subsequent accidents. Lastly, the data of this study do not include the mechanisms of accidents and the severity of injuries. These are important topics for future research, which may help to give a comprehensive view of the drink-driving problem in Vietnam.

## 5. Conclusions

Road collisions related to alcohol is an enormous problem in Vietnam. However, there is a lack of evidence about alcohol use and risk factors among patients suffering from road injuries. This study points out that drink-driving is still a problem in Vietnam. The associated factors with alcohol consumption include socio–economic characteristics—namely gender, employment, and monthly household income. The results also indicate the danger of traffic collisions related to alcohol. These results suggest the need for more stringent laws on drink-driving. In addition, interventions based on smart devices, the internet, and mass media could be feasible solutions for the current context of the drink-driving problem in Vietnam.

## Figures and Tables

**Table 1:** Social-demographic characteristics of respondents.

Characteristics	n	%
**Age group**		
18–30 years	107	29.9
31–40 years	67	16.2
41–50 years	76	18.4
51–60 years	68	16.5
≥60	95	23.0
**Gender**		
Female	253	61.3
Male	160	38.7
**Education**		
Under secondary school	49	11.9
Secondary school	145	35.1
High school	142	34.4
Colleges	46	11.1
≥University	31	7.5
**Marital status**		
Single	93	22.5
Live with spouse/partner	299	72.4
Divorce/Widow	21	5.1
**Occupation**		
Freelance	85	20.6
White-collar worker	25	6.1
Famer/Worker	227	55.0
Student	20	4.8
Others	56	13.6
**Location**		
Rural	60	14.5
Urban	353	85.5
**Role in the traffic collisions**		
Person who caused the crash	40	9.7
Victims	224	54.2
Self-accident	149	36.1
	**Mean**	**SD**
**Age (years)**	45.5	17.0
**Monthly household income (thousand VND)**	8811.9	5325.9

**Table 2:** Alcohol use pattern.

Use Alcohol	n	%
**The frequency of drinking alcohol**		
Never	233	56.4
Monthly or less	63	15.3
2–4 times a month	40	9.7
2–3 times a week	51	12.4
≥Four times a week	26	6.3
**Usual Quantity (Standard alcoholic drinks)**		
1–2	292	70.7
3–4	63	15.3
5–6	25	6.1
7–9	9	2.2
≥10	24	5.8
**The frequency of having six or more drinks**		
Never	262	63.4
Less than monthly	72	17.4
Monthly	41	9.9
Weekly	33	8.0
Daily or almost daily	5	1.2
**Binge drinking**	151	36.6
**Hazardous drinking**	126	30.5
**Consumed alcohol before the collisions**	72	17.4

**Table 3:** Alcohol use according to types of injury and treatment.

Types of Injury and Treatment	Hazardous Drinking					Binge Drinking					Consumed Alcohol Before the Collisions				
	No		Yes		*p*-Value	No		Yes		*p*-Value	No		Yes		*p*-Value
	n	%	n	%		n	%	n	%		n	%	N	%	
Have wounds/Injury															
Flesh-wound	88	30.7	29	23.0	0.11	75	28.6	42	27.7	0.86	95	27.9	22	30.6	0.65
Hand wound	16	5.6	2	1.6	0.07	15	5.7	3	2.0	0.07	18	5.3	0	0	0.04 *
Traumatic brain injury	49	17.1	28	22.2	0.22	40	15.3	37	24.5	0.02 *	53	15.5	24	33.3	0.00 *
Maxillofacial injury	13	4.5	12	9.5	0.05	11	4.2	14	9.3	0.04 *	15	4.4	10	13.9	0.00 *
Spine injury	13	4.5	5	4.0	0.80	12	4.6	6	4.0	0.77	16	4.7	2	2.8	0.47
Chest injury	8	2.8	4	3.2	0.83	7	2.7	5	3.3	0.71	11	3.2	1	1.4	0.40
Fracture	96	33.5	48	38.1	0.36	89	34.0	55	36.4	0.61	119	34.9	25	34.7	0.98
Flesh injury	60	20.9	30	23.8	0.51	59	22.5	31	20.5	0.64	78	22.9	12	16.7	0.25
Number of wound/injury															
0	11	3.8	4	3.2	0.63	11	4.2	4	2.7	0.19	13	3.8	2	2.8	0.19
1	215	74.9	0	71.4		199	76.0	106	70.2		257	75.4	48	66.7	
≥2	61	21.3	32	25.4		52	19.9	41	27.2		71	20.8	22	30.6	
Treatment															
Drug administration	141	49.1	66	52.4	0.54	128	48.9	79	52.3	0.50	166	48.7	41	56.9	0.20
Correction/bundle	44	15.3	12	9.5	0.11	43	16.4	13	8.6	0.03 *	51	15.0	5	6.9	0.07
Flesh surgery	55	19.2	22	17.5	0.68	49	18.7	28	18.5	0.97	63	18.5	14	19.4	0.85
Osteosynthesis	42	14.6	29	23.0	0.04*	38	14.5	33	21.9	0.06	57	16.7	14	19.4	0.58
Tendon joint surgery	6	2.1	2	1.6	0.73	6	2.3	2	1.3	0.49	7	2.1	1	1.4	0.71
Maxillofacial surgery	4	1.4	1	0.8	0.61	2	0.8	3	2.0	0.27	4	1.2	1	1.4	0.88
Others	36	12.5	14	11.1	0.68	32	12.2	18	11.9	0.93	39	11.4	11	15.3	0.36
Number of types of treatment															
0	2	2.7	1	0.8	0.88	2	0.8	1	0.7	0.64	3	0.9	0	0	0.24
1	247	86.1	106	84.1		227	86.6	126	83.4		295	86.5	58	80.6	
≥2	38	13.2	19	15.1		33	12.6	24	15.9		43	12.6	14	19.4	

* *p* < 0.05.

**Table 4:** Factors associated with alcohol abuse.

Factors	Audit Score		Hazardous Drinking		Binge Drinking		Drink Before Accident	
	Coefficient	95% CI	OR	95% CI	OR	95% CI	OR	95% CI
**Gender**								
Male	ref		ref		ref			
Female	−11.13 **	−13.24; −9.02	0.01 **	0.00; 0.05	0.01 **	0.00; 0.03		
**Age group**								
18–30 years	ref		ref				ref	
41–50 years			0.52	0.24; 1.10				
>60 years	−0.85	−2.20; 0.50					0.54	0.21; 1.38
**Education**								
Under secondary school							ref	
Colleges							2.17	0.67; 7.05
**Employment**								
Self-employ								
Famer/Worker	1.13 *	0.00; 2.26	2.32 **	1.35; 3.99	2.09 *	1.19; 3.67		
Student	−3.60 **	−6.17; −1.02			0.42	0.12; 1.55		
**Marital status**								
Single	ref		ref		ref		ref	
Live with spouse/partner			2.61 **	1.40; 4.89	1.53	0.78; 3.01	0.62	0.28; 1.36
Divorce/Widow	−2.34	−5.65; 0.97			0.18	0.02; 1.73		
**Living area**								
Urban	ref							
Rural	−1.30	−2.78; 0.18						
**Monthly household income**								
Lowest	ref		ref				ref	
Lower			1.75	0.82; 3.75			2.09	0.88; 4.99
Moderate	−1.51 *	−2.96; −0.07						
Higher			2.68 **	1.36; 5.27	1.91	0.99; 3.68		
Highest			2.87 **	1.31; 6.26	2.67 *	1.22; 5.85		
**Role in the accident**								
The person caused the accident	ref		ref		ref		ref	
Accident victim	−1.94 *	−3.52; −0.35	0.62	0.37; 1.07	0.58	0.34; 1.00	0.38 **	0.19; 0.78
Self—Accident	−1.15	−2.73; 0.43						
**Injury**								
Flesh wound	−0.89	−2.05; 0.27						
Hand wound	−4.00 **	−7.02; −0.99	0.26	0.05; 1.36	0.25	0.06; 1.10		
Traumatic brain injury							2.40 *	1.08; 5.31
**Treatment**								
Correction/bundle	−1.94 *	−3.54; −0.35	0.58	0.25; 1.32	0.41*	0.18; 0.92		
Other							2.85	0.99; 8.20

** *p* < 0.01, * *p* < 0.05.
